# Incidentally Detected Cardiac Hydatid Cyst: A Rare Case Report

**DOI:** 10.1590/0037-8682-0311-2024

**Published:** 2025-01-27

**Authors:** Fatma Durmaz, Ferhat Kurt, Mesut Özgökçe

**Affiliations:** 1 Van Yuzuncu Yıl University School of Medicine, Department of Radiology, Van, Turkey.

**FIGURE 1:**
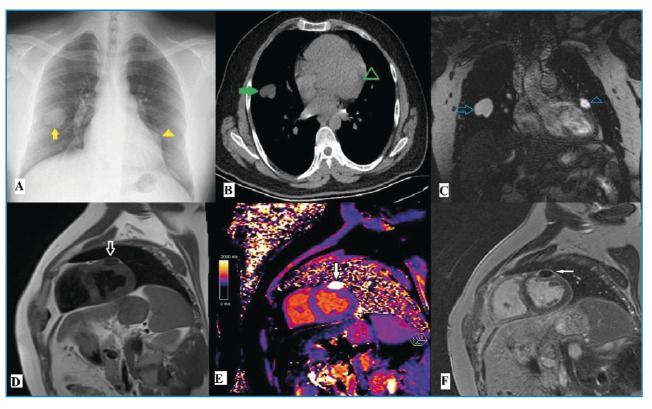
Multimodal imaging of the cardiac hydatid cyst case. **A)** Chest radiograph showing a radiopaque lesion in the right lung (yellow arrow) and bulging of the left cardiac contour. **B)** Axial non-contrast computed tomography demonstrating a cystic lesion in the right lung (green arrow) and a hypoattenuating area in the left ventricular wall. **C)** T2-weighted coronal magnetic resonance (MR) image showing hyperintensity of the lesion in the left ventricular wall (arrowhead). **D)** T1-weighted short-axis MR image displaying the hypointensity of the same lesion (arrow). **E)** T1-mapping MR showing prolonged relaxation time. **F)** Post-contrast cardiac MR demonstrating ring-like enhancement indicative of pericystic fibrotic content.

Hydatid disease, caused by *Echinococcus granulosus*, rarely affects the heart, with less than 2% of cases involving cardiac structures^1^. A 24-year-old man was evaluated for a suspected cardiac mass during a routine examinations for carnitine deficiency. Initial chest radiography revealed a radiopaque area in the right lung and a lobulated contour on the left side of the heart (Figure 1A). Noncontrast chest computed tomography (CT) confirmed a well-defined cystic lesion in the right lung and a focal hypodense area in the left ventricular wall (Figure 1B). Echocardiography performed eight months prior was normal, and serological tests for *Echinococcus* were negative. Cardiac magnetic resonance (CMR) imaging revealed a 26×20 mm lesion in the mid-ventricular anterior wall of the left ventricle that was hyperintense on T2-weighted images (Figure 1C) and hypointense on T1-weighted images (Figure 1D). The lesion showed an increased relaxation time on T1 mapping (Figure 1E) and a relatively thick wall. Early first-pass perfusion CMR indicated low signal intensity with minimal perfusion. Late gadolinium enhancement CMR revealed peripheral enhancement without internal enhancement (Figure 1F). These findings led to a diagnosis of cardiac and pulmonary hydatid cysts, which were confirmed by surgical intervention.

CMR provided detailed insights into lesion morphology and tissue characterization, which are crucial for surgical planning^2^. Owing to the fibrous pericyst content, the peripheral wall of the cyst showed ring enhancement on delayed contrast-enhanced images^3^.

Cardiac echinococcosis should be considered in the differential diagnosis of cystic cardiac lesions. CMR is an essential diagnostic tool for accurate assessments and preoperative planning.
